# 13. Trigeminal Neuralgia

**DOI:** 10.1111/papr.70051

**Published:** 2025-05-19

**Authors:** Bart Jorrit Snel, Steven P. Cohen, Serdar Erdine, Miles R. Day, Jan Van Zundert, Kris Vissers, Jan Willem Kallewaard

**Affiliations:** ^1^ Department of Anesthesiology, Pain and Palliative Medicine Radboud University Medical Center Nijmegen the Netherlands; ^2^ Anesthesiology, Neurology, Physical Medicine & Rehabilitation and Psychiatry and Behavioral Sciences Northwestern University Feinberg School of Medicine Chicago Illinois USA; ^3^ Anesthesiology and Physical Medicine & Rehabilitation Walter Reed National Military Medical Center, Uniformed Services University of the Health Sciences Bethesda Maryland USA; ^4^ Istanbul Pain Center Anesthesiology and Reanimation Department (Algology) Istanbul Turkey; ^5^ Pain Research, the Pain Center at Grace Clinic Texas Tech University HSC Lubbock Texas USA; ^6^ Department of Anesthesiology, Intensive Care, Emergency Medicine and Multidisciplinary Pain Center Ziekenhuis Oost‐Limburg Genk Belgium; ^7^ Department of Anesthesiology, Pain Medicine and Neurology Maastricht University Medical Center Maastricht the Netherlands; ^8^ Department of Anesthesiology, Pain and Palliative Medicine Amsterdam University Medical Center Amsterdam the Netherlands; ^9^ Department of Anesthesiology and Pain Medicine Rijnstate Ziekenhuis Velp the Netherlands

**Keywords:** balloon compression, evidence‐based medicine, gamma knife, microvascular decompression, radiofrequency ablation, stereotactic radiosurgery, trigeminal neuralgia

## Abstract

**Introduction:**

Trigeminal neuralgia (TN) is a disorder characterized by recurrent, unilateral brief electric shock‐like pains, abrupt in onset and termination, limited to the distribution of one or more branches of the trigeminal nerve, and triggered by innocuous stimuli.

**Methods:**

The literature on the diagnosis and treatment of TN was retrieved and summarized.

**Results:**

The diagnosis is made almost entirely based on the patient's history. In classical TN, the neurological examination is typically normal, whereas the exam in secondary TN is focused on surveilling for signs of multiple sclerosis (MS) or a cerebellopontine tumor. The appropriate imaging technique is magnetic resonance imaging (MRI) with contrast of the trigeminal ganglion, which is recommended prior to interventional procedures. The treatment of a patient with TN is a team effort and should always be multidisciplinary, addressing all dimensions of pain. Carbamazepine or oxcarbazepine are first‐line medical treatments. Microvascular decompression (MVD) is the technique of choice for patients without or with minor comorbidities.

Percutaneous procedures for TN are mainly radiofrequency thermocoagulation of the branches of the trigeminal nerve introduced by Sweet and Wepsic in 1965, retrogasserian glycerol injection introduced by Hakanson in 1981, and balloon compression introduced by Mullan and Lichtor in 1983. Radiofrequency treatment is recommended in elderly patients or those with major comorbidities. Other techniques such as stereotactic radiosurgery and pulsed radiofrequency treatment are also discussed.

**Conclusions:**

Recommendations are based on very low‐quality evidence. MVD and radiofrequency are the preferred invasive treatments, although higher‐quality evidence is necessary to better assess the risk–benefit ratios.

## 1 Introduction

This narrative review on trigeminal neuralgia (TN) is an update of the article published in 2009 in the series “Evidence‐based Interventional Pain Medicine According to Clinical Diagnoses.” [1] The previous term for this disorder is Tic Doloreaux, describing the characteristic wince often expressed during a pain paroxysm.

TN is often misdiagnosed or underdiagnosed, but the best estimates at incidence are between 4 and 27 new patients per 100,000 individuals per year [2]. The average age of onset in classical TN is 53–57 years. The incidence of TN is higher among women than men and increases with age [3]. TN is very rare in children and onset before 40 years of age is suggestive of secondary TN [4].

TN most frequently affects the second (maxillary) or third (mandibular) division of the trigeminal nerve, with the right side of the face affected more often than the left side. Bilateral TN is rare and should prompt investigation of the underlying disease. Three types of TN are defined: classical, secondary, and idiopathic, of which the classical type is most common.

Classical TN is caused by intracranial vascular compression of the trigeminal nerve root near its root entry zone in the pons, located in the cerebellopontine cistern. Typically, the vascular culprit is the superior cerebellar artery (SCA), causing lateral dislocation, distortion, flattening, or atrophy of the nerve root, although other arteries or even veins can be the culprit. Anatomical studies have shown that along the proximal portion of the trigeminal nerve, it loses its Schwann cell myelin sheath, in a gradual transition to central myelinization by oligodendroglia. This ‘transition zone’ is particularly vulnerable. Neurovascular conflict causes demyelination at the nerve portion just before it enters the pons.

Cases without vascular compression of the trigeminal nerve are classified as idiopathic TN.

Secondary TN can be caused by tumor compression and multiple sclerosis (MS) [5]. The latter typically causes lesions at the portion of the nerve just distal to its entry into the pons, as is demonstrated in neurophysiological, neuroimaging, and histologic studies [6].

## 2 Methodology

This narrative review is based on the article “Trigeminal neuralgia” published in 2009 [1]. In 2015, an independent company, Kleijnen Systematic Reviews (KSR), performed a systematic review of the literature for the period 2009–2015, based on existing systematic reviews (SRs) and randomized controlled trials (RCTs). For the current article, an updated search was conducted for the period 2015–2022, using “trigeminal neuralgia” and “diagnosis” associated with individual interventional or neurosurgical pain management techniques, such as “decompression surgery, microvascular surgery”, “Gamma Knife radiosurgery” “ablation, radiofrequency catheter”, “radiofrequency”. Additionally, authors could select relevant missing articles from reference lists and other non‐indexed sources.

## 3 Diagnosis

Until recently, there has been no comprehensive, internationally accepted classification of orofacial pain disorders, leading to frequent misdiagnosis and misdirected treatment. In 2020, the first edition of the International Classification of Orofacial Pain (ICOP) was published and is aligned with the International Classification of Headache Disorders, 3rd edition (ICHD‐3; Table 1) and the International Classification of Diseases, 11th revision (ICD‐11) [8].

**TABLE 1:** The ICHD‐3 criteria for the diagnosis of trigeminal neuralgia [7].

Diagnostic criteria (ICHD‐3)
A. Recurrent paroxysms of unilateral facial pain in the distribution(s) of one or more divisions of the trigeminal nerve, with no radiation beyond, and fulfilling criteria B and C
B. The pain has all the following characteristics: lasting from a fraction of a second to 2 min severe intensity electric shock‐like, shooting, stabbing or sharp in quality
C. Precipitated by innocuous stimuli within the affected trigeminal distribution
D. Not better accounted for by another ICOP or ICHD‐3 diagnosis

According to this classification, TN is a disorder characterized by recurrent unilateral brief electric shock‐like pains, abrupt in onset and termination, limited to the distribution of one or more branches of the trigeminal nerve, and triggered by innocuous stimuli. It may develop without an apparent cause or may be a result of another disorder. Additionally, there may or may not be concomitant continuous pain of moderate intensity within the affected division(s). A widely used scheme to measure pain intensity and treatment effect is the Barrow Neurology Institute (BNI) classification, with grade I–V, considering pain intensity and necessity for analgesics [9].

## 4 Physical Examination

Neurological examination is focused on trigeminal sensory function, and signs of MS or a cerebellopontine tumor in the case of secondary TN. In classical and idiopathic TN, the neurological examination typically shows no abnormalities.

## 5 Differential Diagnosis

The posterior scalp, the outer ear (the tragus excluded), and the angle of the mandible are not innervated by the trigeminal nerve [3]. The characteristics of TN are usually typical, with patients reporting stabbing or electric touch‐evoked unilateral pain paroxysms. Many forms of orofacial pain in the region of the trigeminal nerve can be misidentified as TN [2]. These include:

Primary orofacial pain:

– Persistent idiopathic facial pain: The etiology is unknown. Pain is continuous but can be touch‐evoked. It is deep, poorly localized, and does not typically follow the distribution of a dermatomal or nerve innervation.
– Trigeminal autonomic cephalalgia (short‐lasting unilateral neuralgiform headache attacks with autonomic symptoms or with conjunctival injection and tearing (SUNA/SUNCT), paroxysmal hemicrania, cluster headache, or hemicrania continua): pain is accompanied by ipsilateral autonomic symptoms and can alternate sides or rarely be bilateral.

Secondary orofacial pain:

– Trigeminal neuropathy (including posttraumatic and postherpetic neuralgia): usually, there are neurologic abnormalities involving both loss and gain of function in the affected area. Pain can also be continuous, but paroxysms can mimic TN.
– Glossopharyngeal neuralgia: sharp pain paroxysms on the back of the tongue, pharynx, or ear. The painful area can overlap with that of TN. Triggers can include swallowing, sneezing, and coughing.

Odontogenic pain:

– Pulpitis: usually the pain paroxysms last longer, up to several hours.
– Tooth fracture: pain is more circumscribed, triggered when chewing.

The clinician should be aware that percutaneous interventional treatment is less suitable for these differential diagnoses.

## 6 Treatment Options

### 6.1 Conservative Treatments

Carbamazepine or oxcarbazepine continues to be a first‐line medical treatment for primary and secondary TN. The typical dosage of carbamazepine is two‐thirds that of oxcarbazepine, 200 to 1200 mg daily (oxcarbazepine 300 to 1800 mg daily) [10]. These agents are very effective in stabilizing hyperexcited trigeminal neurons, due to blockade of their voltage‐gated sodium channels. This reduces the shock‐like paroxysms, but their effect on continuous pain is limited [3]. Side effects are frequent and include dizziness, ataxia, diplopia, and dose‐related hyponatremia. The most feared side effect of carbamazepine is aplastic anemia, which has an incidence of about 1 in 100,000 but has not been described with oxcarbazepine. Baclofen, a GABA‐B receptor agonist, was shown in a small placebo‐controlled crossover trial to be efficacious for TN and in a recent randomized comparative‐effectiveness study to be non‐inferior to carbamazepine [11, 12]. In other neuropathic conditions, gabapentinoids and antidepressant agents are considered first‐line treatments [13]. In one systematic review, only 2 randomized studies evaluating gabapentin were included (1 with a high risk of bias, the other an unclear risk), prompting the authors to conclude the evidence was insufficient to either support or refuse the use of gabapentin for TN. Unlike carbamazepine, none of these medications are approved for TN [14].

In light of the high recurrence rate for monotherapy in TN, several investigators have examined the effectiveness of combination therapy. Clinical studies have found improved benefit for combination therapy with local anesthetic nerve blocks as add‐ons to both carbamazepine and gabapentin, as well as superiority for electroacupuncture and low‐dose carbamazepine compared to monotherapy and placebo in a 2 × 2 factorial design trial [15, 16].

### 6.2 Invasive Procedures

In general, microvascular decompression (MVD) is the technique of choice for patients without or with minor comorbidities with surgically amenable pathology. In the elderly and patients with major comorbidities, a percutaneous technique is preferred [5].

### 6.3 Neurosurgical Treatment

#### 6.3.1 Microvascular Decompression

Despite an absence of high‐quality evidence, MVD is the most favored neurosurgical procedure for TN when medications are ineffective [3]. After craniotomy and sectioning of the dura mater, the cerebellar hemisphere is shifted to visualize the nerves branching off the pons using a microscope. The vessel that compresses the trigeminal nerve root is kept separated by a small sponge. At 1‐ to 2‐year follow‐up periods, 68% to 88% of patients experience pain relief, with these numbers declining only slightly to 61% to 80% 4 to 5 years post‐procedure [10].

The Cochrane review by Zakrzewska searched for RCTs and quasi‐RCTs through May 2010 on MVD, but no trials met the inclusion criteria for the review [17]. We identified a more recent systematic review investigating surgical MVD for TN [18]. This review evaluated studies through June 2013, with a literature review finding no subsequent RCTs. Therefore, the evidence is based on the observational studies included in the recent systematic review by Xia [18]. Based on 26 observational studies (6847 patients) with an average follow‐up of approximately 3 years, postoperative success rates ranged from 60% to 97%. In 21 of these 26 studies (4469 patients), the offending vessels were reported with a high rate of intraoperative vessel identification (ranging from 77% to 100%). The need to repeat MVD ranged from 0% to 33%. In conclusion, although observational studies of MVD show promising results, the complete risk:benefit ratio remains unclear.

### 6.4 Percutaneous Interventional Treatment

#### 6.4.1 Percutaneous Radiofrequency Treatment

This procedure involves introducing an insulated electrode (usually 20–22‐gauge), with a 2–5 mm active tip, through the foramen ovale under fluoroscopy guidance. The motor and sensory stimulation thresholds are tested to confirm needle position in the distribution of innervation of the desired nerve branch and to minimize masseter involvement. This is outlined in greater detail below. Then, a radiofrequency (RF) treatment is carried out.

In 2020, Mansano et al. published the first randomized double‐blind, sham‐controlled trial evaluating RF lesioning for TN [19]. In this study, 30 participants (15 per group) were followed for 12 months, but failed sham‐treated participants were allowed to cross over after 1 month. This study found a significant and durable pain reduction from day one after RF ablation but was not sufficiently powered to properly evaluate safety.

In 2023 Eskandar et al. conducted a comprehensive review of the current literature on RF treatment [5]. They reviewed 27 retrospective and prospective studies, prioritizing prospective trials and large‐scale cohort studies. In all studies, quality of life improvement was observed. They concluded that a significant improvement in symptoms 1 year after the procedure ranges between 60% and 95%. For isolated V2 neuralgia, a small number of Asian reports investigated the percutaneous treatment via the foramen rotundum [20]. These reports show a slightly better long‐term effect and a lower complication rate of decreased corneal reflex and masticatory weakness. The sample size is very small, and more studies are needed to confirm these findings.

In summary, RF treatment of the Gasserian ganglion seems to have a clinically relevant effect in patients with TN. This procedure is neuroablative, with serious adverse events reported, such as anesthesia dolorosa and sensory loss in the treated branch. In some patients, RF treatment of the Gasserian ganglion may be the best possible treatment option when conservative treatment fails and patients are deemed to be poor candidates for MVD. The duration of effect of RF treatment seems shorter than with MVD, but the risks and invasiveness of MVD justify consideration of RF treatment for patients with serious systemic comorbidities. RF treatment of the Gasserian ganglion should be performed by experts with experience in these procedures after proper education of the patients regarding risks and the likelihood of benefit.

#### 6.4.2 Pulsed Radiofrequency Treatment

This procedure is carried out in the same fashion as percutaneous RF treatment. The delivery of the RF treatment is pulsed, preventing the electrode tip from heating beyond 42^o^C, thereby preserving nerve architecture and eradicating the risk for motor weakness and deafferentation pain, which may occur with an incidence of 0.8% to 3% [21, 22].

RF and pulsed radiofrequency (PRF) were both investigated in the Cochrane review of neurosurgical interventions by Zakrzewska et al. [17]. All trials except for one focused on the delivery of conventional RF treatments. The negative recommendation in the previous guideline for PRF was based on a small RCT comparing the effect of RF of the Gasserian ganglion with PRF in 40 patients [23]. In the PRF group, all participants experienced a return in pain by 3 months. When this group was crossed over to conventional (continuous) treatment, the participants experienced pain control comparable to the group that received RF treatment from the outset. Sensory changes were common in the continuous treatment group, but none were reported in the PRF group. Although this trial was at low risk of bias for randomization and blinding, it was unclear if all patients were followed up for 6 months, and without a control group, it was insufficiently powered for all outcome measures [23].

An additional RCT performed in 60 patients compared short‐duration continuous RF (120 to 180 s), long‐duration continuous RF 240 to 300 s, or PRF for 10 min followed by short‐duration continuous RF [24]. Through a 12‐month follow‐up, all groups experienced significant differences in pain compared to baseline, with no differences between groups. For side effects, the mildest occurred in the short‐duration continuous RF group.

In another RCT with 60 TN patients by Fang et al., the authors found that PRF treatment of the Gasserian ganglion using high voltage resulted in a longer‐lasting effect than PRF treatment at low voltage without compromising safety [25], confirming the findings of a previous retrospective study [26]. High‐voltage PRF treatment appeared to be effective in slightly over two‐thirds of patients 1 year after treatment, though the treatment parameters as described in the manuscript are unclear.

Jia et al. Conducted an RCT comparing high‐voltage PRF of the Gasserian ganglion to peripheral nerve blocks with local anesthetic and steroids plus sham PRF in 134 patients with refractory TN [27]. Excellent or good pain relief was reported in 73% of treatment patients versus 33% who received a nerve block and sham PRF at one‐year follow‐up.

In a retrospective analysis by Chua et al., 36 TN patients underwent PRF treatment of the Gasserian ganglion for 6 min [28]. Excellent pain relief at 2, 6, and 12 months was reported by 76%, 62%, and 56% of patients, respectively.

These results are in line with a larger retrospective study (*n* = 149) by Zipu in 2021 [29]. Of 149 patients who underwent PRF of the Gasserian ganglion for TN, at 2 months, 72% reported at least satisfactory pain relief. Kaplan–Meier analysis of the cumulative recurrence‐free survival was 71% after 1 year and 65% after 2 years.

In 2023, Van Zundert published another retrospective study in 81 patients who underwent PRF treatment of the Gasserian ganglion [30]. Successful pain reduction was reported in 61% of patients, with an average duration of 8 months.

In summary, there may be a role for PRF treatment in TN, as small RCTs and retrospective studies with promising results continue to be published. Although the evidence is of very low quality, significant efficacy and a low complication rate are suggested in comparison to conventional RF. Further investigation is needed regarding the best patients and optimal treatment strategies. The use of high‐voltage PRF could be a promising improvement over standard voltage treatment. At this time, it is not clear what the best voltage selection is. Some authors suggest a dose‐finding study to address this question [31]. They also encourage more basic research studies convincingly demonstrating a non‐destructive effect of high‐voltage PRF.

#### 6.4.3 Percutaneous Balloon Microcompression

This procedure involves decompressing the trigeminal ganglion with a small balloon, inserted via a needle through the foramen ovale under fluoroscopy. This technique may also be applied for TN of the first branch, thereby preserving the corneal reflex. No RCTs have been performed, but a systematic review and meta‐analysis of five retrospective observational studies examining percutaneous interventional treatments in MS‐related TN [32]. No significant differences were found in the immediate pain relief rates between balloon microcompression, glycerol rhizolysis, and RF ablation in MS patients. Balloon microcompression was associated with a higher risk of postoperative mastication weakness compared with glycerol rhizolysis.

#### 6.4.4 Percutaneous Glycerol Rhizolysis

This procedure involves introducing a needle through the foramen ovale under fluoroscopy. In the seated patient with the head flexed, contrast medium is injected into the trigeminal cistern to determine the amount of injectate needed to fill the cistern. The contrast is then aspirated, and the same amount of glycerol is injected [1].

In a Cochrane review of neurosurgical interventions, Zakrzewska et al. included RCTs and quasi‐RCTs, identifying one randomized double‐blind trial comparing steroid rhizotomy with glycerol rhizotomy (0.4 cc of 100% glycerol) [17, 33]. They reported that patients receiving steroid injections returned to baseline levels of pain within 4 weeks, whereas patients receiving glycerol rhizotomy had statistically significant improved outcomes when compared with baseline pain severity. However, the trial was of poor quality, as only 10 patients were included and followed for only four weeks. We did not identify any further RCTs evaluating this procedure. We are aware of at least one nonrandomized study published after the reviews were published [34]. In this small study, excellent pain relief was observed in 57.5% of the glycerol rhizotomy group and either good or excellent relief in 94% of patients. Recurrence was 34% at the end of 12 months and 39% at 5 years. Results were comparable to the RF thermocoagulation group. The authors reported that no major complications were observed during or after procedures, and sensory disturbances were minor and did not require treatment. Overall, the evidence is low for percutaneous glycerol rhizolysis, and the exact balance of benefits and harms compared to other procedures is unknown.

### 6.5 Other Treatment Options

#### 6.5.1 Stereotactic Radiosurgery (Gamma Knife)

This procedure involves noninvasive stereotactic irradiation of a small part of the trigeminal nerve, resulting in a nonselective lesion.

The Cochrane review by Zakrzewska et al. [17] identified only one stereotactic radiosurgery trial [35]. This moderate‐sized (*n* = 87) double‐blind RCT compared the effect of radiation at one or two isocenters in the posterior fossa at a median 26‐month follow‐up. In both groups, there was a significant relapse rate (42%) and incidence of complications (24%), being higher in the group that had two isocenters treated. The authors concluded that for TN, increasing the volume to include a longer nerve length, radiosurgery does not significantly improve pain relief but may increase complications [35].

Evidence on the role of Gamma Knife was extracted from the review of repeat Gamma Knife surgery for recurrent TN by Tuleasca [36]. This review was based on 20 retrospective studies, of which only 4 included more than 40 patients, and 4 had long‐term follow‐up (median range 13.5 to 64.5 months). Effective (> 50%) initial pain relief was reported in a median of 88% (range 60% to 100%) of patients. Median rate of (new) hypoesthesia occurred in 33% (range 11% to 80%). Pain recurrence ranged from 5.3% to 32% with a median of 24 months.

A new search through the end of February 2018 revealed no new SRs, RCTs, or prospective trials relevant to the research question. Stereotactic radiosurgery may have a clinically relevant effect on pain in patients with TN, but the duration of benefit is limited. One major advantage of this treatment is the fact that it is a nonsurgical intervention. No consensus was reached on the type of radiation, duration, or optimal dose. It is unclear which type of patient is most likely to benefit. Hypoesthesia and facial numbness are described in 7.5%–50% of patients, but no serious adverse events have been reported. Based on very low‐quality evidence, it can be concluded that a repeat intervention results in comparable efficacy, but the incidence of side effects (facial hypoesthesia) increases. Considering the risks, this treatment should only be performed by experts based on a “shared decision” model.

## 7 Complications

### 7.1 Microvascular Decompression

Known complications associated with MVD include cerebrospinal fluid leakage, sensory loss, and ipsilateral hearing loss. A systematic review by Xia et al. reported that common, usually transient complications included incisional infection in 1.3% (95% CI: 0.1 to 2.5), facial palsy in 2.9% (95% CI: 0.5 to 6.2), facial numbness in 9.1% (95% CI: 1.3 to 19.6), hearing deficit in 1.9% (95% CI: 0.2 to 3.9) and cerebrospinal fluid leak in 1.6% (95% CI: 0.7 to 2.5) of patients. Mortality was 0.1% (95% CI: 0.02 to 0.2) [18]. An Evidence‐based review by Cohen et al. found an incidence of deafferentation pain between 0.8% and 3% [22].

### 7.2 Percutaneous Radiofrequency Treatment

Percutaneous RF treatment has a fair safety profile. The incidence of serious and life‐threatening complications such as intracranial hemorrhage, intracranial infection, corneal ulcerations, or carotid‐cavernous fistula has significantly decreased with refinements in instruments and technique [5]. In a large retrospective study by Kanpolat et al., no deaths were reported in 1600 patients [37]. Complications included decreased corneal reflex (5.7%), keratitis (0.6%), masseter muscle weakness and paralysis (4.1%), dysesthesia (1%), anesthesia dolorosa (0.8%), transient paralysis of Cranial Nerves III and VI (0.8%), permanent Cranial Nerve VI palsy (0.1%) and cerebrospinal fluid leakage (0.1%). Carotid‐cavernous fistula was observed in one patient (0.06%), and aseptic meningitis in another. In a recent small RCT, Mansano et al. reported that 20% of 30 participants experienced labial herpes simplex reactivation [19]. In 2023, Eskandar and colleagues conducted a comprehensive review of the current literature on RF treatments [5]. They found that lower temperatures seemed to be associated with fewer complications, such as facial numbness, masseter muscle weakness, and diminished corneal reflex. This might also be reflected in the RCT by Mansano et al., which reported a relatively high complication rate using a 75°C lesion. Facial numbness was reported in 40% of the patients 3 months after RF ablation and 18.7% after 12 months. Paresthesia occurred in less than 7% after 12 months. One participant developed masseter muscle weakness. However, it is important to recognize that lesion size, which correlates with “off‐target” tissue damage and side effects, is a function of a combination of lesioning parameters that also include not only temperature but also electrode size, technical parameters (e.g., cooled or tined electrodes), fluid modulation, generator power, and impedance [38, 39].

### 7.3 Pulsed Radiofrequency Treatment

Concerning PRF treatment, no serious complications have been reported in the literature. In the retrospective study by Van Zundert in 81 patients with TN, one patient reported dysesthesia that resolved in 3 months [30].

## 8 Recommendations

The treatment of a patient with essential TN is a team effort that should involve, whenever possible, interdisciplinary care that addresses all dimensions of pain. The various treatment options should be weighed against the risks and considered by all specialist team members. MVD has a longer operation time and postoperative hospital stay than percutaneous treatment, which has to be taken into consideration. Advantages, disadvantages, and risks should be discussed with the patient. For all treatments, the recommendations are based on (very) low evidence. In general, MVD may be a less desirable option in an elderly patient with multiple comorbidities, whereas percutaneous RF treatment can be a more viable alternative in high‐risk surgical candidates. In a setting of a “shared decision making” model, PRF treatment can also be considered. Although the evidence for efficacy is less robust than for conventional RF treatment, the complication rate is lower. Our recommendations are listed in Table 2.

**TABLE 2:** Evidence‐based recommendations for the interventional treatment of trigeminal neuralgia.

Technique	Level of evidence	Evidence	Recommendation	Strength of recommendation
MVD	Very low	There is very low‐quality evidence or the efficacy of MVD in the treatment of TN.	MVD can be used as first‐line therapy in younger patients and those without serious comorbidities who have TN of vascular origin that is refractory to conservative treatment.	Very weak
RF treatment	Low	There is low‐quality evidence that RF treatment of the Gasserian ganglion reduces pain in patients with TN for at least 6 months.	RF treatment of the Gasserian ganglion can be used in patients suffering from TN refractory to conservative treatment and for whom surgical intervention (MVD) is not an option.	Weak
PRF treatment	Low	There is low‐quality evidence that PRF treatment of the Gasserian ganglion is effective in the treatment of TN.	PRF (standard and high voltage) treatment of the Gasserian ganglion for patients suffering TN should be considered only in poor surgical candidates in select cases based on a shared decision model.	Very weak
Stereotactic radiosurgery	Very low	There is very low‐quality evidence for the effectiveness of stereotactic radiosurgery in reducing pain in patients with TN.	Stereotactic radiosurgery should be considered for patients suffering TN refractory to conservative treatment who are not candidates for other therapies.	Very weak

Abbreviations: MVD, microvascular decompression; PRF, pulsed radiofrequency; RF, radiofrequency; TN, trigeminal neuralgia.

## 9 Clinical Practice Algorithm

**FIGURE 1:**
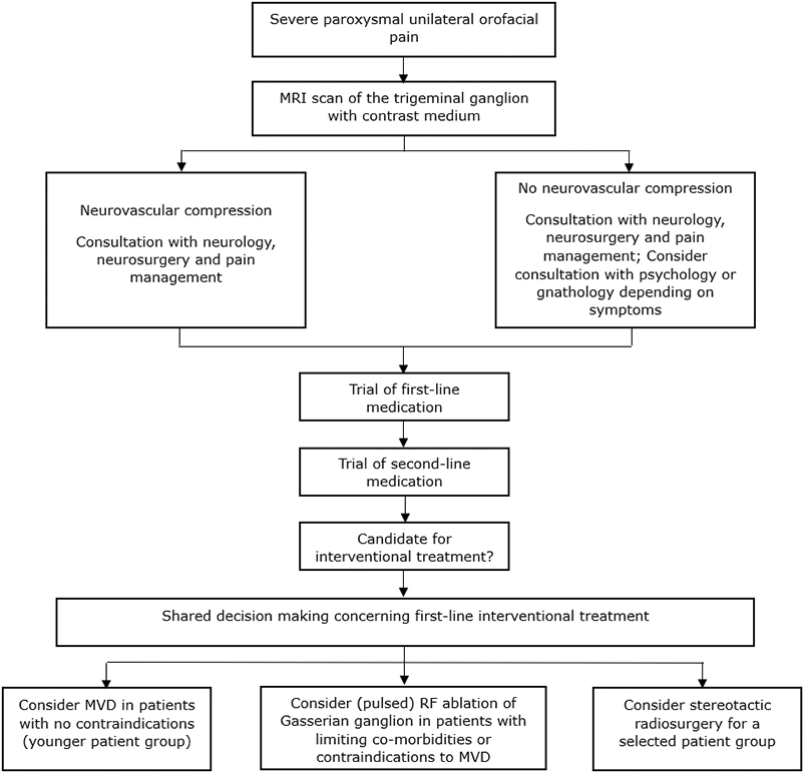
Algorithmic approach for the assessment and treatment of trigeminal neuralgia. MRI, magnetic resonance imaging; MVD, microvascular decompression; RF, radiofrequency.

A practice algorithm for the management of TN is illustrated in Figure 1.

## 10 Technique

### 10.1 (Pulsed) Radiofrequency Treatment Technique

The procedure is performed using fluoroscopy. The patient lies supine, and monitoring is performed in accordance with standard American Society of Anesthesiologists (ASA) guidelines, with nasal oxygen delivery. Most clinicians perform this block under moderate sedation, waking up the patient when the needle is in place for stimulation. The use of local anesthetic is controversial.

**FIGURE 2:**
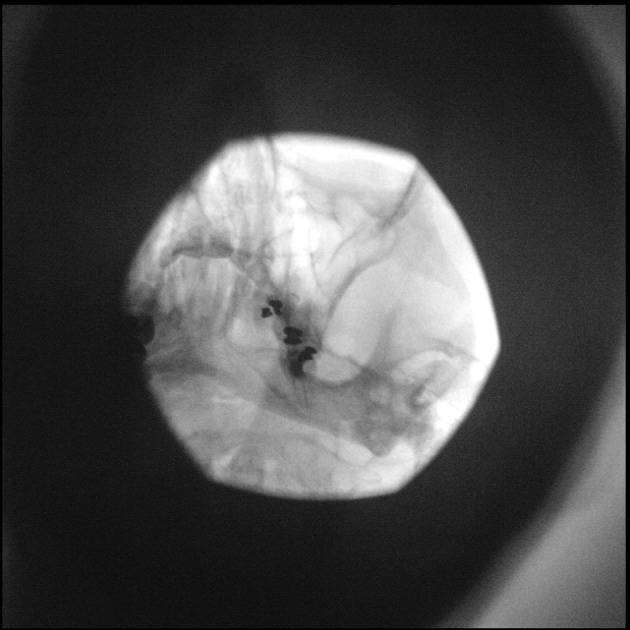
Fluoroscopy image of percutaneous (pulsed) RF treatment of the Gasserian ganglion, oblique submental view. Image courtesy of K.T.E. Olde Dubbelink.

**FIGURE 3:**
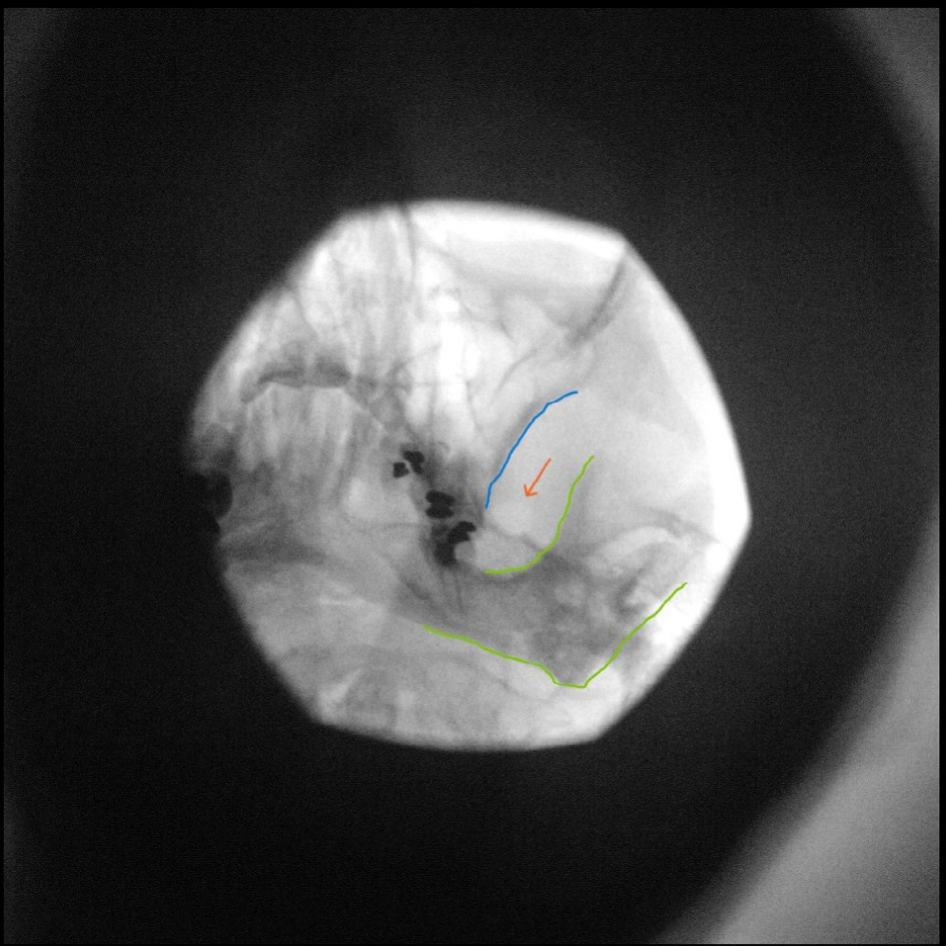
Fluoroscopy image of percutaneous (pulsed) RF treatment of the Gasserian ganglion, oblique submental view. Lines: Maxilla (blue), mandibula (green), foramen ovale (orange). Image courtesy of K.T.E. Olde Dubbelink.

The head of the patient is taped and a posteroanterior (PA) submental view is obtained. The degree of caudad angulation depends on the head position. A good anatomical starting point is a 45° angle with the coronal plane of the face. The C‐arm is slowly tilted obliquely to the painful side, usually around 15°. The foramen ovale will appear caudally and laterally to the maxillary sinus and medially to the ramus mandibulae (Figures 2 and 3).

An RF ablation cannula, with a 2–5 mm active tip, is in a coaxial view. The entry point is usually 1 to 3 cm lateral to the corner of the mouth. If the mandibular fibers are targeted, the entry point will be more medial, and the lateral part of the foramen ovale is targeted. If the ophthalmic fibers are targeted, the entry point is more lateral, and the medial part of the foramen ovale is targeted.

**FIGURE 4:**
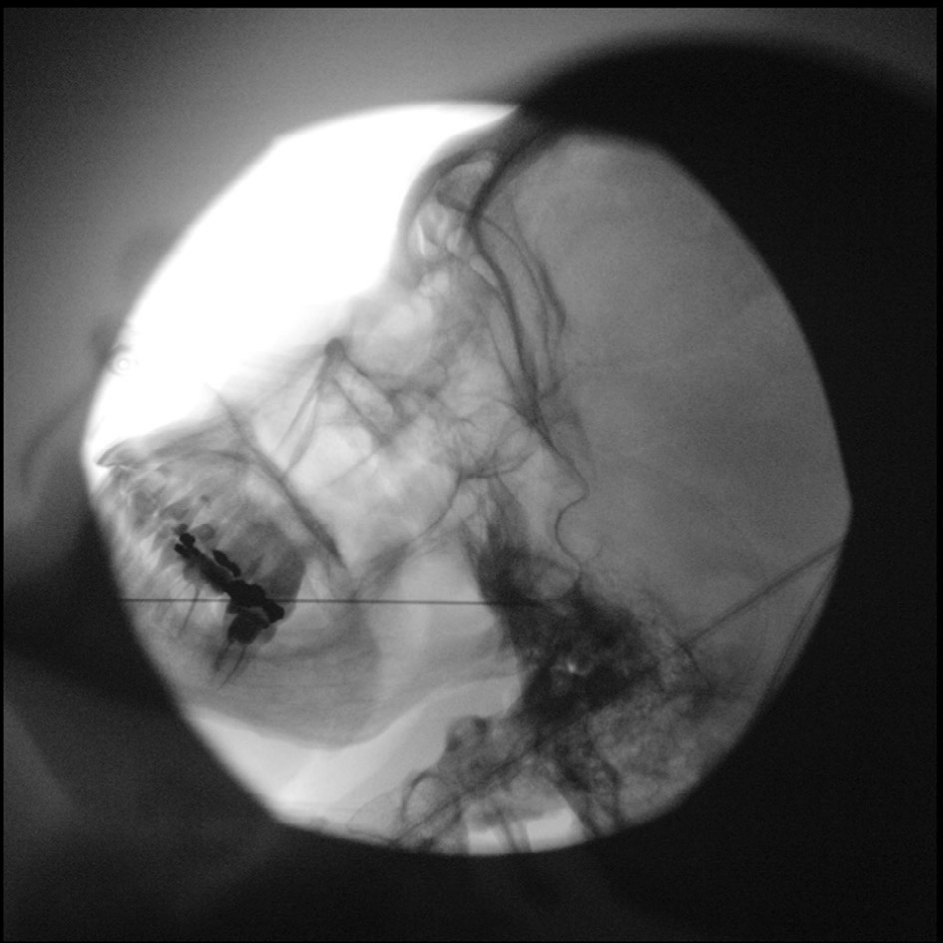
Fluoroscopy image of percutaneous (pulsed) RF treatment of the Gasserian ganglion, lateral view. The needle is advanced through the foramen ovale. Image courtesy of K.T.E. Olde Dubbelink.

Continuing in a coaxial view (tunnel vision) the needle is advanced to the foramen ovale, while checking for possible accidental penetration of the oral mucosa before entering the intracranial space (this can be done by intraoral palpation or visual inspection). A lateral view is obtained to confirm needle depth. The needle tip should not pass beyond the clivus in this view. If the needle passes the foramen ovale, it is engaged in the trigeminal ganglion, and a stimulation test can be carried out (Figure 4).

After discontinuation of sedation, a motor stimulation test at 2 Hz will produce masseter twitches. Slowly, the needle can be advanced until the twitches fade. Motor stimulation should occur < 0.6 V. A sensory stimulation test is carried out at 50 Hz at up to a 1 V setting to confirm the needle is advanced into the part of the ganglion corresponding with the desired sensory (nerve) distribution.

Sedation can then be resumed if needed. RF technique: treatment at 60° for 60 s is applied. Assess the patient again for hypoesthesia, corneal reflex, and stimulation thresholds. If there are no abnormalities, another radiofrequency treatment can be done at 65° for 60 s. The same assessment is carried out, and optionally a final treatment can be carried out at 70° for 60 s; some practitioners elect to slightly adjust the needle (e.g., rotate a curved active tip) to maximize the capture rate. PRF treatment is applied with the temperature limited to 42° for at least 240 s. Assessment of the patient for neurological abnormalities is not necessary.

## Ethics Statement

The authors have nothing to report.

## Consent

The authors have nothing to report.

## Conflicts of Interest

The authors declare no conflicts of interest.

## Data Availability

Data sharing not applicable to this article as no datasets were generated or analyzed during the current study.
